# Coexpression of natural killer cell antigens by T-cell large granular lymphocytes in hydroa vacciniforme lymphoproliferative disorder and the involvement of Vδ1 + epithelial-type γδT cells

**DOI:** 10.1007/s12185-023-03599-7

**Published:** 2023-05-03

**Authors:** Yoji Hirai, Keiji Iwatsuki, Takahide Takahashi, Tomoko Miyake, Yuki Nakagawa, Shogo Tanimoto, Yoshio Kawakami, Shin Morizane

**Affiliations:** 1 Department of Dermatology, Okayama University Graduate School of Medicine, Dentistry, and Pharmaceutical Sciences, 2-5-1 Shikata-Cho, Kita-Ku, Okayama, 700-8558 Japan; 2 Division of Dermatology, Fukushima Rosai Hospital, 3 Numajiri, Uchigo Tsuzura-Machi, Iwaki, 973-8403 Japan; 3 Division of Medical Support, Okayama University Hospital, 2-5-1 Shikata-Cho, Kita-Ku, Okayama, 700-8558 Japan

**Keywords:** Hydroa vacciniforme, Chronic active EBV disease, Large granular lymphocyte, γδT-cell, NK-cell antigen

## Abstract

Hydroa vacciniforme lymphoproliferative disorder (HV-LPD) is a cutaneous variant of chronic active Epstein–Barr virus disease. We examined the coexpression of T- and natural killer (NK)-cell antigens in five patients with classic HV (cHV) and five with systemic HV (sHV). T-cell receptor (TCR) repertoire analysis was performed with high‑throughput sequencing. All five cHV patients had increased γδT cells (> 5%), whereas five sHV patients showed γδT- and αβT-cell dominance in two patients each, and a mixture of abnormal γδT and αβT cells in one. Circulating CD3 + T cells expressed CD16/CD56 at 7.8–42.3% and 1.1–9.7% in sHV and cHV, respectively. The percentage of CD16/CD56 + T cells was higher in the large granular lymphocyte or atypical T-cell fractions in sHV, but no TCR Vα24 invariant chain characteristic of NKT cells was detected. Considerable numbers of CD3 + cells expressing CD56 were observed in sHV skin infiltrates. Of the circulating γδT cells tested, TCR Vδ1 + cells characteristic of the epithelial type of γδT cells were dominant in two sHV cases. Thus, atypical αβT and γδT cells in HV-LPD can express NK-cell antigens, such as CD16 and CD56, and Vδ1 + epithelial-type γδT cells are a major cell type in some HV-LPD cases.

**Supplementary Information:**

The online version contains supplementary material available at 10.1007/s12185-023-03599-7.

## Introduction

Chronic active Epstein–Barr virus disease (CAEBV) is characterized by a broad disease spectrum encompassing the cutaneous and systemic forms [1, 2]. The cutaneous forms include hydroa vacciniforme lymphoproliferative disease (HV-LPD) and severe mosquito bite allergy (SMBA) [3]. HV-LPD includes two clinical subtypes: a benign and self-limited disease, designated as classic HV (cHV), and a more aggressive disease termed systemic HV (sHV). The latter subtype is characterized by facial edema with HV-like skin lesions associated with transient systemic symptoms such as fever and liver damage and often overlaps with SMBA. Unlike CAEBV (ICD-O coding: 9725/1), or “systemic” CAEBV in the WHO classification, patients with sHV have neither sustained organ involvement, except for the typical skin lesions on examination, nor infectious mononucleosis-like symptoms persisting for > 3 months, one of the major diagnostic criteria for CAEBV [4]. In the clinical course over the years, most patients with sHV progress to fatal outcomes with internal organ involvement and hematological diseases comparable with those of CAEBV [5–10].

HV-LPD patients often have a dominant EBV + T-cell clone, together with the other minor EBV + T/NK and B-cell subsets [11, 12]. Typically, patients with cHV have increased percentages of EBV + γδT cells (> 5% of lymphocytes) in the peripheral blood [5, 6], whereas most SMBA patients have increased percentages of EBV + natural killer (NK) cells (> 30% of lymphocytes) [5, 6, 13]. Patients with sHV are divided into two groups: the γδ T-cell-dominant type and the αβT-cell-dominant type [5]. The γδT-cell-dominant type is observed in younger individuals and shows a favorable prognosis, whereas the αβT-cell-dominant type may also occur in adults, often associated with increased numbers of EBV + T-cell large granular cells (LGLs) [14], and take a fatal outcome in many cases [5].

The disease subtype and the responsible lymphocyte subset seem to be closely related; there have been some controversies and matters of concern. For instance, although HV-LPD is usually a disease mediated by EBV + T cells, the NK-cell type of HV-LPD has also been reported [15–17]. In such cases, we should consider the overlapping of SMBA and HV-LPD in the same patient [18]. Furthermore, we should remember the fact that activated CD3 + T cells are capable of expressing NK-cell antigens such as CD16 and CD56 [19].

In HV-LPD cases, the cell lineage of proliferating γδT cells remains unclear, although a previous flow cytometry analysis has revealed an increase in Vδ2 + γδT cells, a dominant subset of circulating γδT cells in healthy individuals [20, 21]. In contrast, lymphoma cells of Vδ1 + γδT-cell origin are often observed in cutaneous γδT-cell lymphoma arising from the outer layer of skin, hepatosplenic T-cell lymphoma, and type II enteropathy-associated T-cell lymphoma (γδT-cell type) [22]. So far, the involvement of Vδ1 + epithelial-type γδT cells has not been elucidated in the pathogenesis of HV-LPD.

This study was conducted to confirm that proliferating CD3 + γδT or αβ T cells in HV-LPD cases can express NK-cell antigens, such as CD16 and CD56, excluding the possibility of natural killer T (NKT) cell lineage. Additionally, we further attempted to determine possible cell lineage of proliferating γδT cells in HV-LPD, based on the results of TCR repertoire analysis using high-throughput sequencing,

## Materials and methods

### Patients and blood/skin samples

We examined patients with cHV who fulfilled the following criteria [6, 7]: (1) presence of repetitive vesiculopapular eruptions on exposed areas, including the face, lips, cheeks, and extensor surfaces of the hands and arms; (2) presence of histological features of reticulated degeneration of the epidermis or blister formation associated with dense lymphocytic infiltration; (3) presence of EBV-encoded small nuclear RNA (EBER) in skin lesions; and (4) exclusion of hereditary photosensitivity disorders.

In contrast, patients with sHV presented with one or more of the following clinical and histopathologic findings in addition to the EBER + HV-like eruptions: (1) high-grade fever (> 37.5 °C), (2) liver damage, (3) ulcerative indurated lesions, and (4) edematous swelling of the cheeks, eyelids, ears, and lips.

We enrolled five patients with sHV and five patients with cHV in the study. No patients had NK lymphocytosis, and EBER + CD3 + cell infiltration was confirmed in the skin biopsy specimens. The control group was composed of five patients with adult T-cell leukemia/lymphoma (ATLL) and two patients with leukemic cutaneous T-cell lymphoma (CTCL). The following three cell types were defined as NK cells by flow cytometry: 1) sCD3 − CD16 − CD56 + , 2) sCD3 − CD16 + CD56 − , and 3) sCD3 − CD16 + CD56 + cells. Using high-throughput sequencing, cells harboring an invariant TCR chain of Vα24-Jα18/Vβ11 were determined as natural killer T (NKT) cells [23]. Their blood and skin biopsy samples had been obtained for diagnostic use, and in this study, we reevaluated these patients’ previous laboratory test results or used the remnants of the samples. This study was approved by the Institutional Review Board of the author’s university (#1610–008) in accordance with the 1975 Declaration of Helsinki. Written informed consent was obtained from all patients enrolled in the study.

### Flow cytometry

We performed a flow cytometric analysis of peripheral blood mononuclear cells (PBMCs) from five patients with sHV, five with cHV, five with ATLL, and two with leukemic CTCL. We used our routine panel of conjugated antibodies for flow cytometry: anti-sCD3, CD4, CD7, CD8, CD16, CD25, CD30, CD45, CD56, TCR PAN αβ (clone: IP26A), TCR PAN γδ (clone: IMMU510), and HLA-DR antibodies (Beckman Coulter, Indianapolis, IN, USA). A Navios instrument (Beckman Coulter) was used for all multicolor flow cytometry, and the data were analyzed using Kaluza software (Beckman Coulter).

### T-cell receptor repertoire analysis with high-throughput sequencing

Total RNA was isolated from PBMCs of a patient with αβT-cell-dominant sHV (case 3 in Table 1) using the RNeasy Plus Universal Mini Kit (Qiagen, Hilden, Germany). The amount and purity of the RNA samples were measured using an Agilent 2100 bioanalyzer (Agilent Technologies, Palo Alto, CA, USA). Unbiased amplification of TCR genes and subsequent high-throughput sequencing were subsequently performed as described [24].

**Table 1:** HV patients enrolled in this study and the immunophenotype of atypical cells

Cases	Sex	Age (onset) y	Clinical subtype	Dominant lymphocyte subset	γδT (%)	αβT (%)	NK (%)	CD56 + cells (%) in sCD3 + fr	CD16 + cells (%) in sCD3 + fr	CD16/CD56 + cells (%) in sCD3 + fr	Atypical lymphocytes: LGLs fr. or cell groups with an aberrant immunophenotype	Outcomes
1	Male	15 (13)	sHV	γδT	7.2	61.4	6.6	5.1	7.8	NA	LGL fr.: CD3dim + , γδT + (88.3%), CD8 + / − , DR + , CD16 + (82.5%), and CD56 + (34.7%) in CD3 + fr	Alive
2	Female	28 (17)	sHV	γδT	13.1	59.8	8	8.6	6.8	NA	CD3high + γδT + , CD8 + / − , CD16 + (27.5%), CD56 + (23.7%) in CD3 + fr	Alive
3–1	Female	28 (23)	sHV	αβT	NA	CD4 + (78.9), CD4 + CD8 + (14.6)	1.46	42.3	NA	NA	CD3 + , CD4 + , CD8 − , αβT + (78.9%) CD3 + , CD4 + , CD8 + , αβT + (14.6%), CD56 + (44%) in CD3 + fr	Died of HPS and myocardial and intestinal infiltration
3–2		32 (32)	sHV CAEBV	Mixed	3.55	55.7	38.2	NA	NA	38.2	LGL fr.: CD3 + , αβT + (69.6%), γδT + (17.3%), CD16/CD56 + (60.3%) in CD3 + fr	
4	Male	74 (74)	sHV CAEBV	αβT	1	CD8 + (82.5), CD4 + (15.3)	9.3	NA	NA	34.4	CD3 + CD8 + (84.5%), abT + (97.6%), CD56 + (37.8%)	Died of CAEBV
5	Female	78 (60 s)	sHV	Mixed	7.9	73.8	7.8	14.2	NA	NA	Gr.1: CD3dim + , TCR- (66.9%), CD56 + (21.6%) Gr.2: CD3 + , αβT + (95.4%), CD56 + (69.8%) Gr.3: CD3high + , γδT + (94.8%), CD56 + (100%)	Died of intestinal bleeding of unknown cause
6	Male	3 (3)	cHV	γδT	8.7	61.8	7.6	3.38	1.76	NA	CD3high + , γδT + , DR +	Alive
7	Male	10 (4)	cHV	γδT	5.1	69.4	7.4	3.8	NA	NA		Alive
8	Male	6 (5)	cHV	γδT	7.6	66.1	22.7	1.1	NA	NA		Alive
9	Female	18 (6)	cHV	γδT	9.9	70.3	9.8	1.2	NA	NA	CD3 + , CD8dim + , γδT +	Alive
10–1	Male	6 (5)	cHV	γδT	36	42.2	4.67	NA	NA	9.7	CD3 + , CD4 − , CD8 + / − , γδT +	Alive
10–2		6 (5)	cHV	γδT	24.4	48.1	6.86	NA	NA	9.56	CD3 + , CD4 − , CD8 + / − , γδT +	

*HV* hydroa vacciniforme, cases 1–5: *sHV* systemic HV, cases 6–10: *cHV* classic HV, *CAEBV* chronic active EBV disease, *sCD3* surface CD3, *LGLs* large granular lymphocytes, *NA* not available, *HPS* hemophagocytic syndrome, NK cells were defined as (1) sCD3 − CD16 − CD56 + , (2) sCD3 − CD16 + CD56 − , and (3) sCD3 − CD16 + CD56 + cells. The term of CD16/CD56 indicates CD16 and/or CD56

### Immunostaining

For immunostaining, we used formalin-fixed, paraffin-embedded tissue sections. After deparaffinization and peroxidase blocking, the sections were stained with mouse monoclonal anti-human CD3, CD4, CD8, and CD56 (Dako Japan, Tokyo, Japan). Slides were then incubated with ChemMate Envision polymer (Dako Japan). The target proteins were detected using a diaminobenzidine tetrahydrochloride solution.

## Results

### Dominant lymphocyte subsets in systemic and classic hydroa vacciniforme

Clinical backgrounds and outcomes of all HV-LPD patients enrolled in the present study have been summarized in Table 1.

The percentages of γδ T cells in the peripheral blood lymphocytes were increased (5.1%–36%) in all five patients with cHV, whereas no notable abnormalities in cell count and cytology were observed among the other lymphocyte subsets (Table 1). Our previous study showed that EBER + cells were observed in approximately 50–80% of circulating T cells in HV-LPD patients, including cHV and sHV, and EBER + cells were mainly detected in the proliferating T-cell fractions [6].

Of the five blood samples obtained from sHV patients, we observed that the percentages of γδT cells were increased in three patients: cases 1, 2, and 5 at 7.2, 13.1, and 7.9% in the peripheral blood lymphocytes, respectively (Table 1). Flow cytometry revealed an absence of abnormalities in the αβT - and NK-cell fractions in cases 1 and 2, and they were, thus, classified as having the γδT -cell-dominant type. Case 5 was diagnosed as a mixed type of γδT and αβT cells based on the coexistence of αβT cells with an aberrant immunophenotype. In the remaining two patients with sHV, CD3 + CD4 +  αβT cells were predominant in case 3 (93.5%), the fraction of which also contained CD4+CD8+ cells (14.6%), while CD3 + CD8 +  αβT cells were increased up to 82.5% in case 4. These two patients died of a progressive illness.

### Coexpression of NK-cell antigens, CD16, and CD56 by circulating CD3 + T cells

**Fig. 1:**
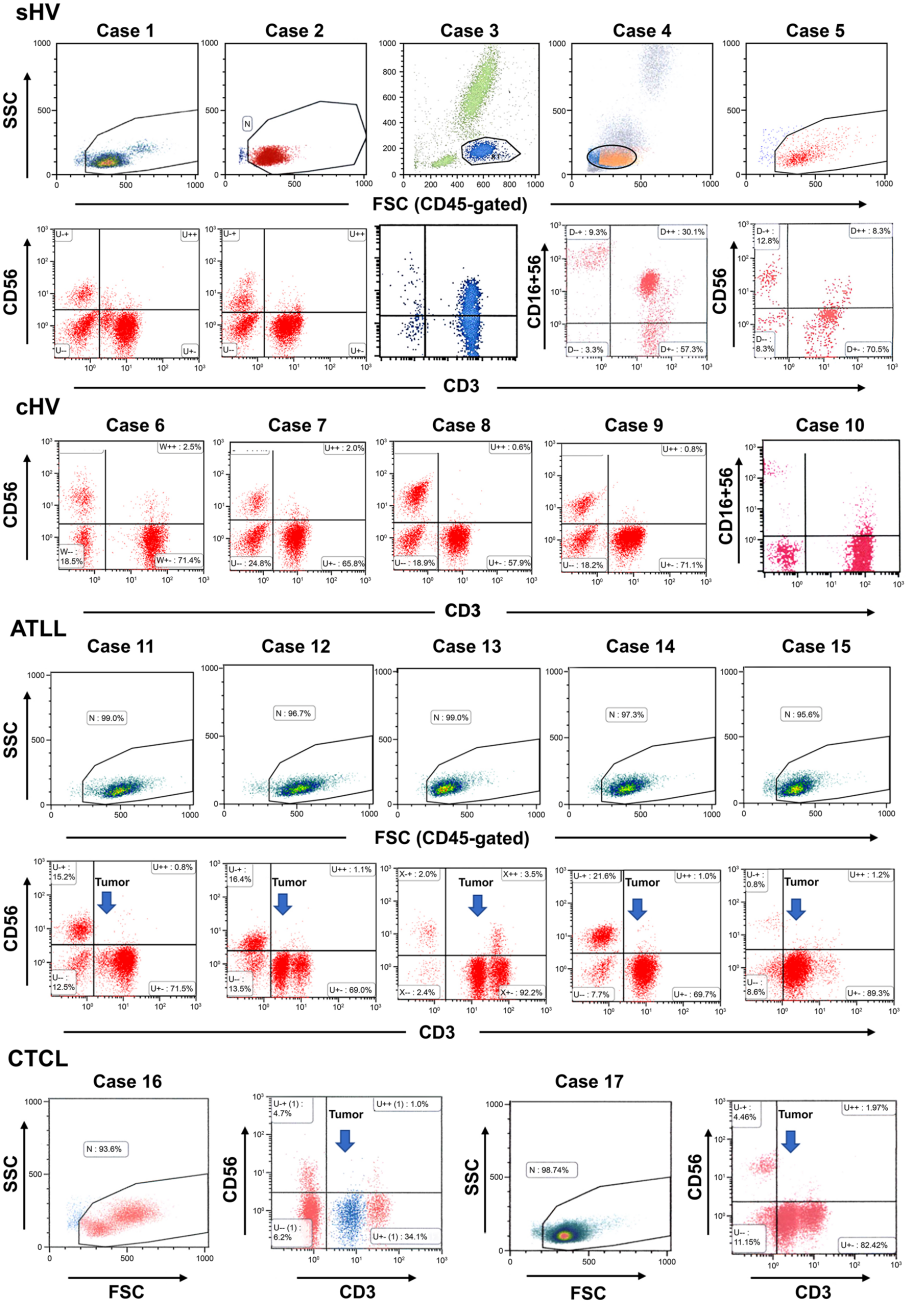
Coexpression of CD16/CD56 by CD3 + cells in HV-LPD, ATLL, and CTCL. Arrows indicate tumor cell fractions

**Fig. 2:**
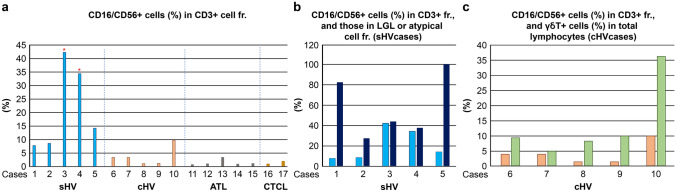
Percentages of CD16/CD56 + cells in the HV-LPD and control groups, **A** CD3 + T cells expressed CD16/CD56, ranging from 7.5 to 42.3% in HV-LPD. Some patients with cHV also expressed CD16/CD56 in the CD3 + cell fractions. In contrast, tumor cells in ATL and CTCL expressed CD16/CD56 in less than 1.2%. (**B**, **C**) large granular lymphocytes (LGLs) of sHV expressed CD16/CD56 in higher percentages

Flow cytometric analysis revealed that CD3 + T cells in sHV expressed the NK antigens CD16 and/or CD56 (CD16/CD56) at a lower fluorescence intensity (CD16/CD56dim +) than the true NK cells (Fig. 1). In the sHV patients, CD3 + T cells coexpressed CD16/CD56, ranging from 7.8% to 42.3% in the whole CD3 + fractions (Table 1; Fig. 2A). In the two patients with the αβT-cell-dominant sHV, the percentages of CD16/CD56 + cells were extremely high: 42.3% in case 3, and 34.4% in case 4. The five patients with cHV had CD16/CD56dim + cells in the whole CD3 + cell fractions, ranging from 1.1% to 9.7% (Fig. 1 and 2A). In contrast, the tumor cells of the patients with ATLL and CTCL expressed CD16/CD56 less than 1.2%.

**Fig. 3:**
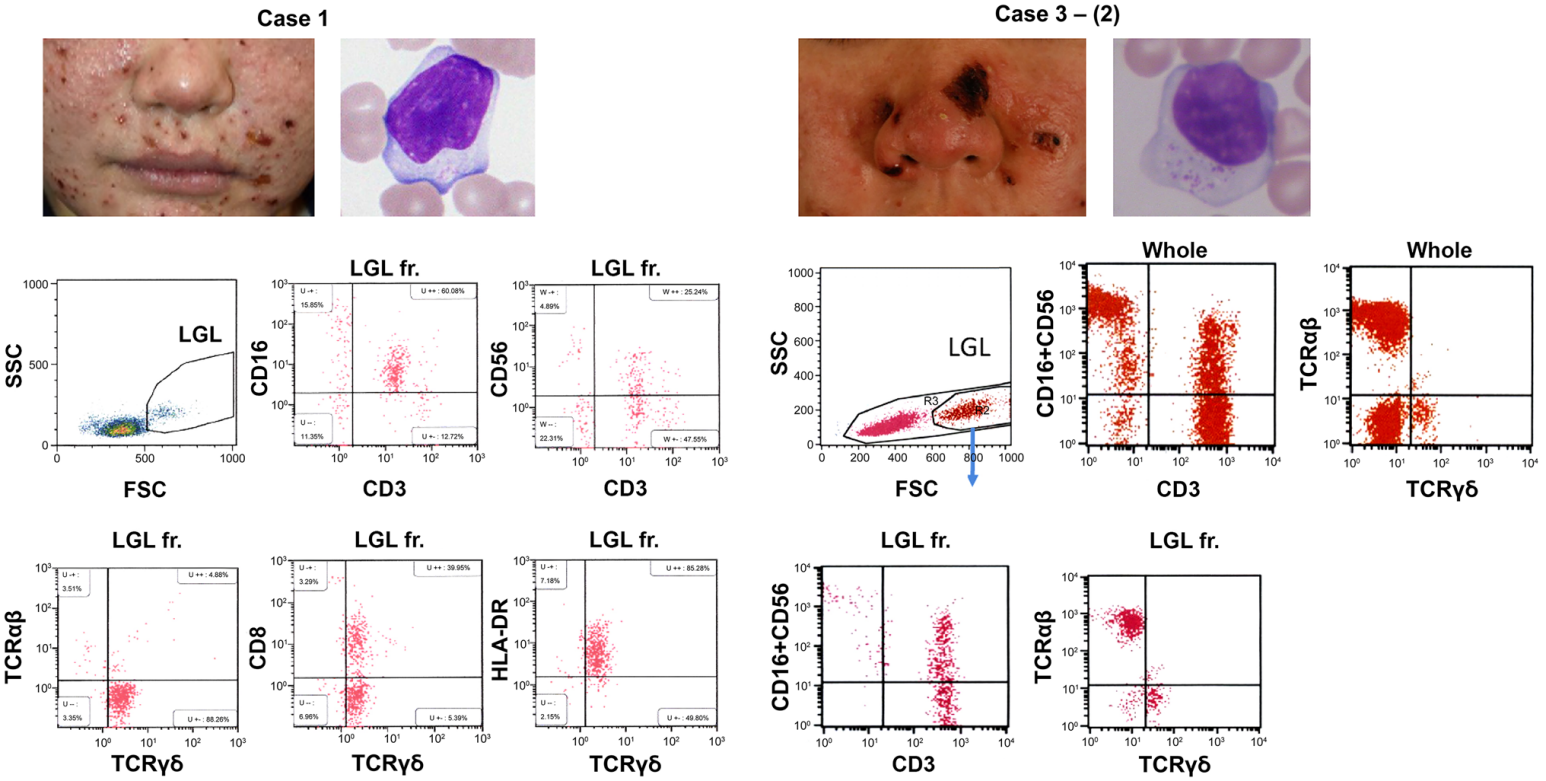
Percentages of CD16/CD56 + cells in LGL fractions. Blood samples from cases 1 and 3-(2) contained CD3 + T-cell type LGL (insets); mainly γδT cells in case 1 and a mixture of αβT and γδT cells in case 3. The T-cell LGLs coexpress CD16/CD56 at 82.5 and 54.0%, respectively

We examined the percentages of CD16/CD56 + cells in the LGL fractions (> 500 in forward scatter value) of the sHV patients because LGLs are a cytological hallmark of EBV + cytotoxic T/NK cells in HV-LPD and SMBA [13, 14]. When LGL fractions were not clearly separated by flow cytometry, the percentages of CD16/CD56 + cells were examined in atypical T-cell fractions with aberrant immunophenotypes (Table 1). The results revealed that the percentages of CD16/CD56 + cells increased in the LGL (cases 1 and 3) or the atypical T-cell fractions (cases 2, 4, and 5) than those in the whole CD3 + cells (Fig. 2B). For instance, CD16dim + cells were observed in 82.5% of the CD3 + LGLs in case 1, in which most LGLs were of the γδT-cell lineage, and frequently coexpressed CD8 and an activation marker, HLA-DR (Fig. 3). In the second blood sample obtained from case 3, CD16/CD56dim + cells were detected in 60.3% of the CD3 + LGLs containing αβT-cell (69.6%) and γδT-cell LGLs (17.3%) (Fig. 3).

### Detection of CD3 + CD56 + cells in the skin infiltrates in the T-cell-dominant systemic hydroa vacciniforme

**Fig. 4:**
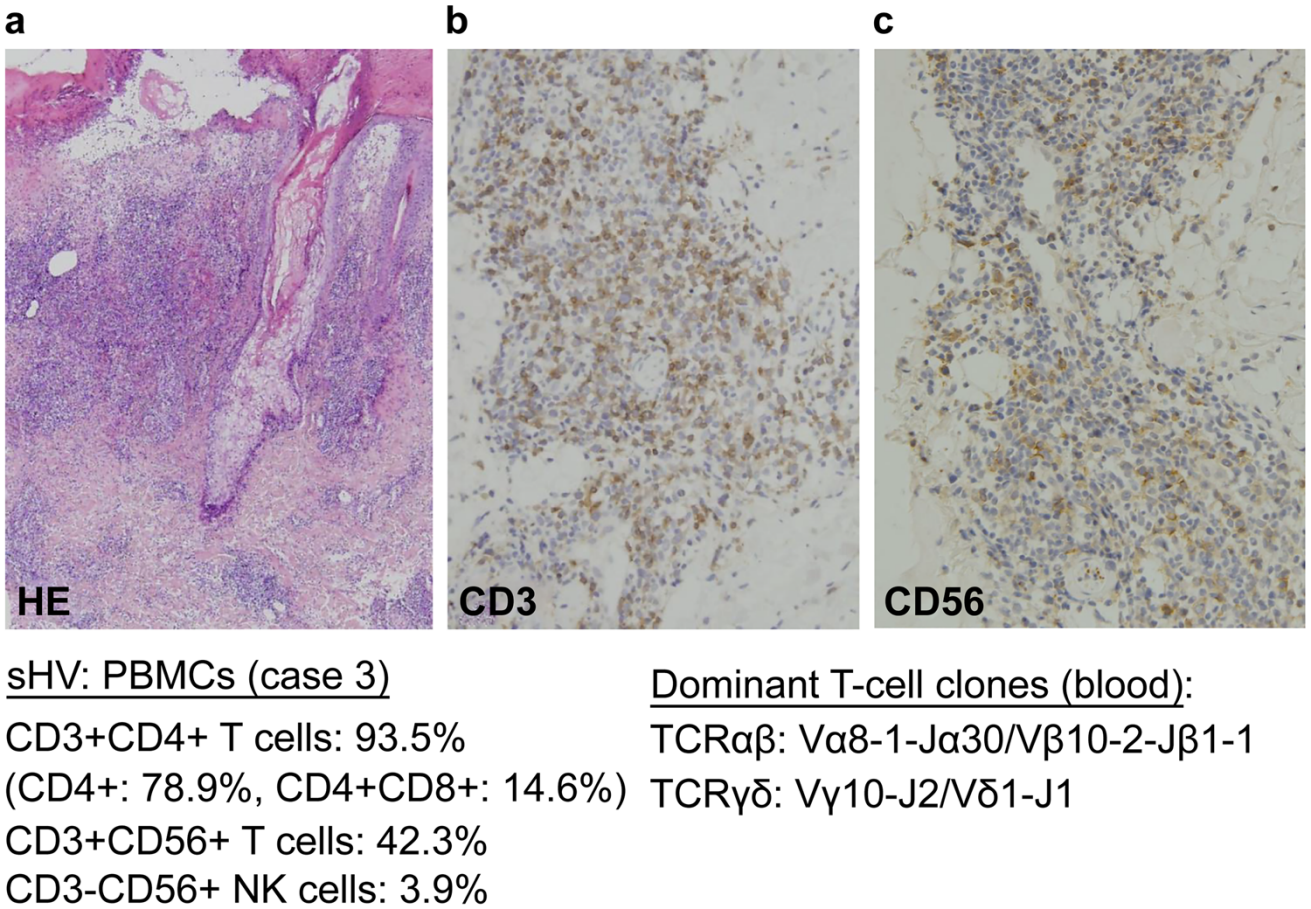
Presence of CD56 + cells in the dermal infiltrates of sHV (case 3) and TCR usage of the dominant T-cell clones in the blood. **A** HV-like skin lesion (HE), **B** CD3 + cells, and **C** CD56 + cells. The peripheral blood mononuclear cells (PBMCs) contain 93.5% CD3 + CD4 + T cells, including CD4 + CD8 + cells (14.6%). The CD3 + cells coexpress CD56 in 42.3%. Two dominant T-cell clones are present in the PBMCs; a major αβT-cell clone with Vα8–1-Jα30/Vβ10–2-Jβ1–1 (genetic code: TRAV8-1-TRAJ30/TRBV10-2-TRBJ1-1, 43.0 and 59.1% in a total read count, respectively) and a minor γδT-cell clone with Vγ10-Jγ2/Vδ1-Jδ1 (genetic code: TRGV10-TRGJ2/TRDV1-TRDJ1, 83.1 and 75.2%, respectively). No T-cell clone with Vα24, characteristic of human invariant NKT cells, was detected

A considerable number of CD56 + cells were observed in the CD3 + CD4 + cells in the dermal infiltrates of sHV skin lesions (case 3 in Table 1; Fig. 4). This patient’s blood contained CD3 + CD4 + T cells at 93.5%, and CD3 + CD56dim + T cells were observed at 42.3% in the CD3 + cell fraction. CD3-CD56 + NK cells were present in only 3.9% of the blood.

### T-cell receptor usage of αβT- and γδT-cell clones in the peripheral blood

**Fig. 5:**
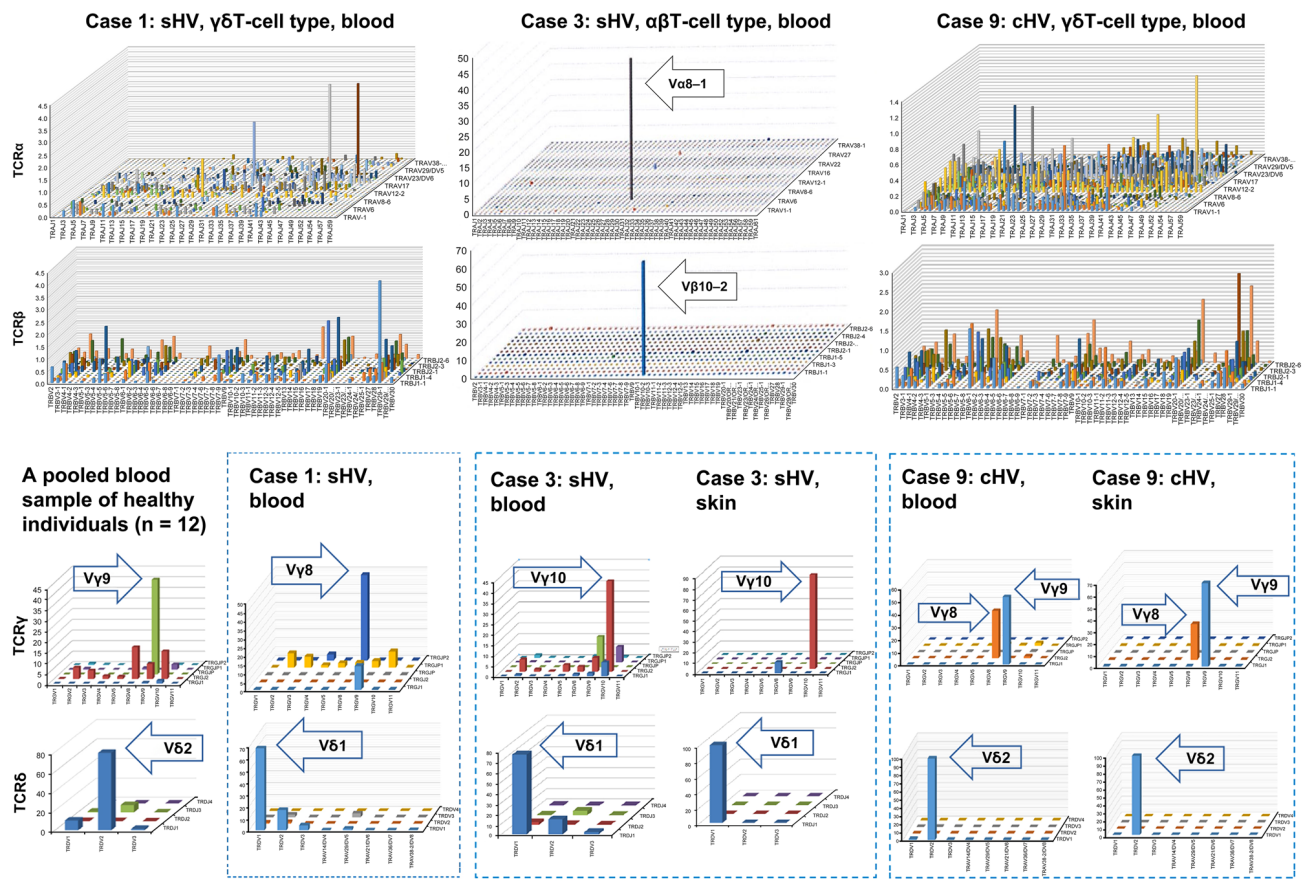
The αβT-cell clone is detected in the αβT-cell type sHV (case 3); its diversity is evident in the γδT-cell type (cases 1 and 9). Epithelial Vδ1 + T cells were found in cases 1 and 3. A common subset of another Vγ9/Vδ2 + T-cell clone was detected in cHV (case 9)

In case 3 (αβT-cell-dominant sHV), a major αβT-cell clone carried TCRαβ, composed of Vα8–1-Jα30/Vβ10–2-Jβ1–1 (genetic code: TRAV8-1-TRAJ30/TRBV10-2-TRBJ1-1; Fig. 4). There was no T-cell clone bearing the TCR of Vα24-Jα18/Vβ11 characteristic of human invariant NKT cells [23]. In addition to the major αβT-cell clone, another γδT-cell clone harboring the epithelial type of Vδ1 + TCR (Vγ10-Jγ2/Vδ1-Jδ1, genetic code: TRGV10-TRGJ2/TRDV1-TRDJ1) was detected, whereas a pooled sample from healthy individuals had a usual dominant γδT-cell clone with a TCR composed of Vγ9-JγP/Vδ2-Jδ1. The same Vδ1 + γδT-cell clone was more selectively observed in the HV skin lesion (Fig. 5).

Another sHV patient (case 1) had a single dominant peak of γδT cells expressing Vδ1 + TCR (Vγ8-JγP2/Vδ1-Jδ1), which is also the epithelial type of γδT cells (Fig. 5). In contrast, one cHV patient (case 9) had two major γδT-cell clones with Vδ2 + TCR (Vγ9-JγP/Vδ2-Jδ1 and Vγ8-Jγ1/Vδ2-Jδ1) in blood and skin lesions.

## Discussion

Our analyses revealed that in sHV patients, some cell populations of CD3 + αβT and γδT cells expressed NK-cell antigens (i.e., CD16 and CD56), although the expression intensity of the NK-cell antigens was lower than that of true CD3-CD56 + NK cells. The percentages of CD16/CD56dim + cells were increased in the T-cell LGL fractions in sHV (Figs. 2, 3), in which latent EBV infection is usually confirmed [13, 14]. In our cHV patients, the CD3 + T cells also contained small percentages of CD16/CD56dim + cells. Although EBV presence was not directly confirmed on the surface of CD3 + T and γδT cells, most NK-like T cells may correspond to EBV + γδT cells, as EBER + cell counts and EBV DNA loads are increased in the γδT-cell fractions of cHV [6, 19, 25, 26]. Furthermore, EBER + cells are primarily observed in the γδT-cell fraction using flow cytometric analysis on surface immunophenotypes and EBER^5^ (Supplementary Fig. S1).

In contrast, CD3 + CD4 + ATLL cells carrying an oncogenic virus (i.e., human T-cell leukemia virus type-1 [HTLV-1]) expressed CD16/CD56 at < 1.2%. Therefore, latent EBV infection, but not HTLV-1 infection, might be a driving force for inducing the NK-cell antigens CD16 and CD56 on the CD3 + T cells. Unfortunately, however, we have no direct evidence that EBV + γδT or αβT cells induce CD16/CD56 expression. Without EBV infection, CD56 is expressed in various cytotoxic T-cell lymphomas or leukemias, including primary cutaneous CD8 + aggressive epidermotropic cytotoxic T-cell lymphoma, mycosis fungoides with a cytotoxic immunophenotype, subcutaneous panniculitis-like T-cell lymphoma, type II enteropathy-associated T-cell lymphoma, hepatosplenic T-cell lymphoma, and T-cell LGL leukemia [27–29].

Two possible mechanisms have been considered regarding the coexpression of T- and NK-cell antigens: activation-induced expression of CD16/CD56 by T cells and specific cell lineage (such as invariant NKT cells) that can induce T- and NK-cell antigens. Previous studies have reported that when monocyte-depleted human PBMCs were cultured with interleukin (IL)-12 and IL-2, the surface CD3 + CD56 + cells with cytotoxic activity were selectively expanded [30]. Indeed, proinflammatory cytokines, such as tumor necrosis factor-α, are highly elevated in the blister fluid of HV [23], and constitutively activated STAT3, with subsequent production of cytokines, is found in EBV + T/NK cells in CAEBV patients [31].

Alternatively, CD3 + CD16/CD56 + cells might be of human invariant NKT-cell lineage with a TCR of Vα24-Jα18/Vβ11 [23]. However, the results of our TCR repertoire analysis revealed no use of Vα24-Jα18/Vβ11 in the dominant αβT-cell clones (case 3; Fig. 4); therefore, CD3 + CD16dim + and CD3 + CD56dim + cells in sHV are not of invariant NKT-cell lineage. That patient (case 3) had another γδT-cell clone harboring Vδ1 + TCR in the blood characteristic of the epithelial type of γδT cells. It is intriguing to note that two different clones (i.e., a dominant αβT-cell clone and a minor γδT-cell clone) were detected in the same patient. This observation might account for the overlapping of various clinical manifestations in sHV patients and the replacement of dominant clones during the patient’s clinical course.

Our TCR sequencing identified a dominant γδT-cell clone derived from Vδ1 + cells in two sHV patients (cases 1 and 3): Vγ8-JγP2/Vδ1-Jδ1 and Vγ10-Jγ2/Vδ1-Jδ1, respectively, whereas two distinct Vδ2 + clones were detected in one cHV patient (case 9) examined Vγ9-JγP/Vδ2-Jδ1 and Vγ8-Jγ1/Vδ2-Jδ1, in which the former is a major γδT clone in the peripheral blood from healthy individuals [20]. The latter observation is consistent with the previous one that Vδ2 + γδT cells were predominant in CAEBV patients associated with HV-LPD [21, 26, 32]. Our results, however, showed that a Vδ1 + epithelial-type γδT-cell clone was predominant in the sHV skin lesion as well as the patient’s blood. Given the fact that neoplastic cells of epidermotropic γδT-cell lymphomas are mainly of a Vδ1 + γδT-cell lineage [22], the similar γδT cells in HV-LPD might be capable of epidermal homing to form the skin lesions.

To determine the cell types infiltrating the HV-LPD lesions, the interpretation of the presence of CD3 + CD56 + cells in the tissue should be considered. CD3 + CD56 + cell infiltration is usually sparse in HV-LPD lesions [33] but is sometimes observed considerably enough to enable a diagnosis of NK-type HV-LPD [15–17]. In the cutaneous lesions of αβT-cell-dominant sHV (case 3), our immunostaining method detected CD3 + CD56 + cells in the dermal infiltrates. Since approximately one-half of the patient’s CD3 + αβT cells coexpressed CD16 and CD56, we believe that the presence of CD3 + CD56 + cells in skin lesions does not provide a clue for NK-cell origin. However, since the EBV + NK cells were isolated from HV-like eruptions, we cannot deny the possibility that some EBV + NK cells exist in the skin infiltrates [34].

Measuring EBV DNA load in PBMCs is useful for the diagnosis of CAEBV, but it does not help predict the prognosis [11]. In the present study, the percentages of CD3 + CD16dim + and CD3 + CD56dim + cells were higher in the sHV group than in the cHV group, indicating a favorable prognosis (Fig. 2). All five patients with cHV were alive during the observation period; however, three (cases 3, 4, and 5) of five patients with sHV succumbed to hemophagocytic lymphohistiocytosis (HLH), myocardial and intestinal infiltration of EBV + T cells, and intestinal bleeding of unknown cause, respectively, and serious systemic diseases comparable with CAEBV. Our previous prognostic analysis on 19 patients with HV-LPD, including these cases, indicated that three of eight (38%) sHV patients died during the observation periods ranging from 4 to 9 years (median; 7 years), while all 11 cHV patients were alive, although two patients (18%) showed disease progression[7].

Since many patients with sHV progress to CAEBV and some patients with CAEBV may present with HV-like vesicles during the clinical course, it is difficult to clearly differentiate those subtypes upon first examination. Patients with HV-like skin lesions and transient systemic symptoms not fulfilling the criteria for CAEBV are classified into systemic HV and observed carefully [4].

Among the sHV group, two patients with fatal sHV of the αβT-cell-dominant type exhibited much higher percentages of CD3 + CD16dim + and CD3 + CD56dim + cells in their CD3 + T-cell fractions. Therefore, in HV-LPD patients, the percentage of CD3 + cells harboring the NK-cell antigens might be a surrogate marker for the tumor burden.

## Conclusion

In conclusion, our observations indicate that atypical αβT and γδT cells with an LGL feature or aberrant immunophenotypes coexpress NK-cell antigens, such as CD16 and CD56, but these cells are distinct from the invariant NKT-cell lineage. Furthermore, some HV-LPD patients have a dominant epithelial-type γδT-cell clone bearing Vδ1 + TCR.

## Supplementary Information

Below is the link to the electronic supplementary material.

Supplementary file1 Supplementary Fig. S1. (A) (Left) EBER+ cells (purple) in the PBMCs from a cHV patient are conformed by EBER in situ hybridization analysis. (B) (Right) EBER+ cells (red) are mainly observed in the γδT-cell fraction using flow cytometric analysis of surface immunophenotypes and EBER. These data are reproduced from our article in JID 2012 (TIF 1136 KB)

## Data Availability

Data can be used only with the permission of the research institution, hospital and the first author.
