# Supplementary figures and images for: Coexpression of natural killer cell antigens by T-cell large granular lymphocytes in hydroa vacciniforme lymphoproliferative disorder and the involvement of Vδ1 + epithelial-type γδT cells
Source: Int J Hematol. 2023 May 3;118(1):54–64. doi: 10.1007/s12185-023-03599-7 (PMC10285018; doi:10.1007/s12185-023-03599-7)

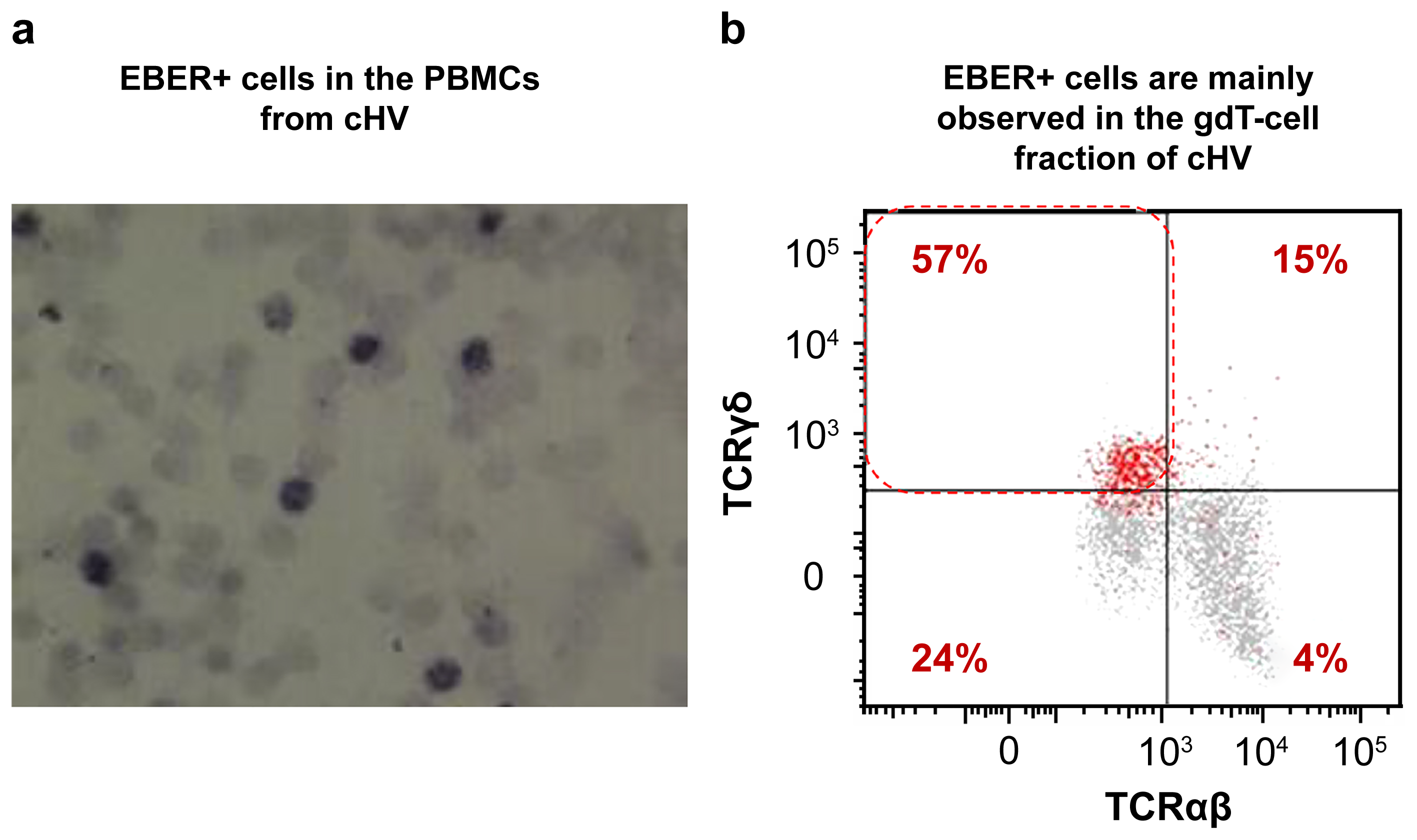

Supplement: Supplementary file 1 — Supplementary file1 Supplementary Fig. S1. (A) (Left) EBER+ cells (purple) in the PBMCs from a cHV patient are conformed by EBER in situ hybridization analysis. (B) (Right) EBER+ cells (red) are mainly observed in the γδT-cell fraction using flow cytometric analysis of surface immunophenotypes and EBER. These data are reproduced from our article in JID 2012 (TIF 1136 KB) [file 12185_2023_3599_MOESM1_ESM.tif]
